# EDTA-modified carbapenem inactivation method (eCIM) for detecting IMP Metallo-β-lactamase–producing *Pseudomonas aeruginosa*: an assessment of increasing EDTA concentrations

**DOI:** 10.1186/s12866-020-01902-8

**Published:** 2020-07-20

**Authors:** Maxwell J. Lasko, Christian M. Gill, Tomefa E. Asempa, David P. Nicolau

**Affiliations:** 1 Center for Anti-Infective Research and Development, Hartford Hospital, 80 Seymour Street, Hartford, CT 06102 USA; 2 Division of Infectious Diseases, Hartford Hospital, Hartford, CT USA

**Keywords:** Carbapenemase, Resistance, Zinc-dependent, Metallo-β-lactamase, *Pseudomonas aeruginosa*

## Abstract

**Background:**

Prompt identification of carbapenemase-harboring organisms is valuable in informing therapeutic and infection-control measures. The modified carbapenem inactivation method (mCIM) and EDTA-modified carbapenem inactivation method (eCIM) are inexpensive and easy to interpret phenotypic tests endorsed by the Clinical and Laboratory Standards Institute (CLSI) for the detection of carbapenemase-harboring Enterobacterales. Only mCIM is endorsed by CLSI for detecting carbapenemase-harboring *Pseudomonas aeruginosa*. eCIM’s ability to delineate serine and metallo-β-lactamases (MBL) could be advantageous in areas prevalent with carbapenemase-harboring *P. aeruginosa*. A recent assessment of mCIM/eCIM on MBL-harboring *P. aeruginosa* demonstrated high eCIM sensitivity for NDMs and VIMs but not for IMP-producers. Therefore, this study aimed to determine whether increasing EDTA concentrations would enhance eCIM sensitivity for a collection of IMP-harboring *P. aeruginosa* isolates*.*

Twenty-six IMP-harboring *P. aeruginosa* isolates were utilized. For test validation, additional *P. aeruginosa* isolates harboring NDM (*n* = 3), VIM (n = 3), KPC (*n* = 8), wild-type (*n* = 1), and Enterobacterales isolates harboring IMP (*n* = 6) and NDM (n = 1) were assessed. The mCIM test was conducted as outlined by CLSI. Simultaneously, the eCIM test was performed with the standard 5 mM EDTA concentration and doubling EDTA concentrations: 10 mM, 20 mM, and 40 mM.

**Results:**

Concentration-dependent improvement was observed among the IMP-harboring *P. aeruginosa* with eCIM sensitivities at 0, 31, 85, and 100% respectively*.* Remaining Enterobacterales and *P. aeruginosa* responded concordantly with their genotype at the standard 5 mM eCIM concentration, with doubling EDTA concentrations providing no greater sensitivity.

**Conclusion:**

Combination of mCIM and an eCIM with a 40 mM EDTA concentration appropriately capture IMP-harboring *P. aeruginosa* without sacrificing test utility for other carbapenemase-harboring isolates.

## Background

*Pseudomonas aeruginosa* is a frequent cause of nosocomial infections and exhibits high intrinsic resistance to many common antimicrobials [1, 2]. Furthermore, horizontal transfer of genetic elements including carbapenemase genes can confer augmented resistance limiting treatment options [3]. Thus, the detection of carbapenemase-producing organisms is paramount for treatment decisions as well as infection control [4]. The Clinical and Laboratory Standards Institute (CLSI) endorsed modified carbapenem inactivation method (mCIM) and EDTA-modified carbapenem inactivation method (eCIM) have emerged as a combination phenotypic test to detect and differentiate between serine and metallo-based carbapenemases for Enterobacterales [5]. Their use of inexpensive products and easy to interpret results are favorable for clinical laboratories with limited resources.

A recent study assessing the utility of tandem mCIM/eCIM against a variety of clinical metallo-β-lactamase (MBL)-producing *P. aeruginosa* isolates demonstrated high eCIM sensitivity for NDMs and VIMs but not for IMP-producers [6]. The poor sensitivity in differentiating IMP-harboring *P. aeruginosa* isolates as metallo-based enzymes mirrors similar findings among IMP-harboring Enterobacterales [7]. The authors demonstrated 5 mM provided optimal sensitivity compared to the initially investigated 0.1 mM [7]. Therefore, this current study aimed to determine whether increasing EDTA concentrations would enhance eCIM sensitivity for a contemporary collection of IMP-harboring *P. aeruginosa* isolates.

## Results

### mCIM/eCIM evaluation

**Fig. 1:**
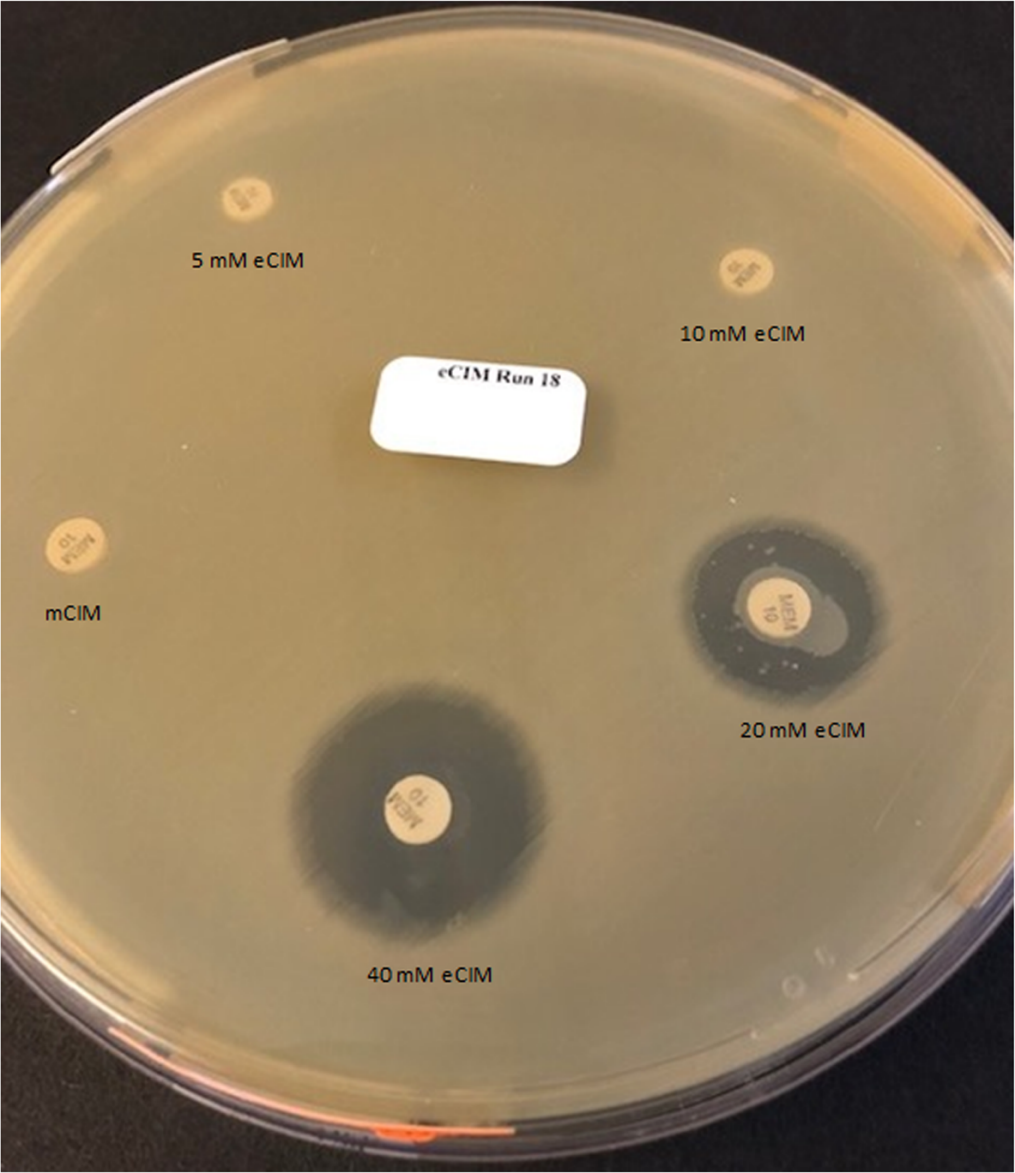
mCIM/eCIM results for an IMP-48 harboring *P. aeruginosa*

Concordance between eCIM phenotypic tests and IMP-harboring *P. aeruginosa* isolates was observed in 0% (0/26), 35% (9/26), 85% (22/26), and 100% (26/26) of isolates at EDTA concentrations of 5 mM, 10 mM, 20 mM, and 40 mM respectively. Figure 1 illustrates a mCIM and eCIM test result for an IMP-harboring *P. aeruginosa* isolate. To complement percent concordance, eCIM zone sizes were recorded at each concentration for all utilized isolates, demonstrating an EDTA concentration-dependent effect for IMP-harboring *P. aeruginosa* isolates only. Notably, there were no major differences in zone of inhibition diameter among different eCIM concentrations once an isolate’s phenotypic result matched its genotype with an exception of one isolate. An IMP-48 harboring isolate’s zone of inhibition diameter increased from 12 mm to 26 mm as seen in Fig. 1. Moreover, 3 of the 4 *P. aeruginosa* isolates harboring IMP-48 were false negative in the presence of 20 mM EDTA, requiring the 40 mM EDTA concentration to achieve 100% sensitivity (Table 1). NDM- and VIM-harboring *P. aeruginosa* and Enterobacterales isolates were eCIM true positive at the standard eCIM concentration of 5 mM, with doubling EDTA concentrations providing no greater sensitivity. As expected, all 8 serine-carbapenemase-harboring *P. aeruginosa* resulted in true negative eCIM results. Higher concentrations of EDTA had no detrimental effect on the growth or sensitivity of the test for any isolate.

**Table 1:** Sensitivity and Specificity of eCIM at Respective EDTA Concentrations

	IMP-harboring *P. aeruginosa* Only	All Evaluable Isolates^a^	
eCIM Concentration	Sensitivity [*n* = 26] (95% CI)	Sensitivity [*n* = 39] (95% CI)	Specificity [n = 8] (95% CI)
5 mM EDTA	0% (0–15%)	33% (19–50%)	100% (63–100%)
10 mM EDTA	35% (17–56%)	56% (40–72%)	100% (63–100%)
20 mM EDTA	85% (65–96%)	90% (76–97%)	100% (63–100%)
40 mM EDTA	100% (87–100%)	100% (91–100%)	100% (63–100%)

^a^ All evaluable isolates consist of *P. aeruginosa* harboring IMP, NDM, VIM, and KPC, as well as Enterobacterales harboring IMP and NDM. WT was not included in eCIM sensitivity or specificity analysis

### Establishment of controls

The three evaluable control isolates were tested interday over 8 days. Observed eCIM results were 100% concordant with their genotype. Thus, *P. aeruginosa* 27853, *P. aeruginosa* #0441, and *P. aeruginosa* #0444 were utilized as controls throughout the study.

## Discussion

At present, the standard eCIM test is a simple and inexpensive method for differentiating serine and metallo-β-lactamase activity in Enterobacterales. With an increased EDTA concentration of 40 mM, the eCIM test offers high sensitivity in differentiating between metallo- and serine- carbapenemase production among a diverse collection of *P. aeruginosa*. Improvements in eCIM sensitivity to IMP-producing *P. aeruginosa* are essential, given high prevalence rates, second only to VIM [8–10].

Similar improvements in eCIM sensitivity among Enterobacterales were observed in a study by Sfeir and colleagues [7]. An increase from 0.1 mM to 5 mM EDTA concentration increased eCIM sensitivity from 75 to 100%. Of note, at 0.1 mM, all three IMP-positive Enterobacterales resulted as false negative, which was resolved at 5 mM. Another study evaluating the eCIM test with IMP-producing Enterobacterales demonstrated 55% (6/11) eCIM sensitivity with IMP-producing isolates, with the authors hypothesizing a higher EDTA concentration may be warranted [11]. Yamada and colleagues further evaluated several metal chelators, including EDTA, in conjunction with mCIM testing against IMP-positive Enterobacterales obtained from Japanese hospitals (*n* = 93). An increase from 5 mM to 10 mM EDTA among IMP-positive Enterobacterales enhanced eCIM sensitivity from 79.6 to 98.9%, again demonstrating an EDTA concentration-dependent improvement in sensitivity [12]. Notably, sub-genotypes were not reported in the aforementioned studies, and based on our findings with IMP-48, variability in the eCIM test performance may be enzyme-subtype specific and warrants additional investigation and reporting in future studies.

This study has limitations worth noting. No *P. aeruginosa* isolates evaluated in this study harbored both a metallo-β-lactamase and a serine carbapenemase. While rare, these dual carbapenemase-harboring isolates would likely result in a false negative eCIM interpretation regardless of EDTA concentration and remains an inherent limitation of the eCIM test. A multi-center validation study using an increased EDTA methodology is warranted.

## Conclusion

In summary, we observed an EDTA concentration-dependent improvement in eCIM sensitivity among IMP-harboring *P. aeruginosa*, providing an important contribution to optimization of this phenotypic test. At a 40 mM EDTA concentration, the eCIM provides high sensitivity for differentiating between metallo-dependent and serine carbapenemase-producing *P. aeruginosa* and provides clinical laboratories a reliable and accurate phenotypic screening assay.

## Methods

### Bacterial isolates

Forty-eight clinical isolates were utilized including 26 IMP-harboring *P. aeruginosa* isolates. For test validation, additional *P. aeruginosa* isolates harboring NDM (*n* = 3), VIM (n = 3), KPC (*n* = 8), wild-type (*n* = 1) as well as Enterobacterales isolates harboring IMP (*n* = 6) and NDM (n = 1) were assessed. Fourteen were acquired from the Centers for Disease Control and Food and Drug Administration Resistance Bank (CDC and FDA-ARB) and the remaining from the Center of Anti-Infective Research and Development isolate library. Isolates were previously categorized by PCR or whole genome sequencing for the detection of β-lactamase producing genes. Evaluated enzyme subtypes harbored among evaluated *P. aeruginosa* isolates included IMP (− 1, − 6, − 7, − 10, − 18, − 48, − 62), VIM (− 2, − 5), NDM (− 1), and KPC (− 2, − 5). Evaluated Enterobacterales isolates harbored IMP (− 1, − 4, − 8) and NDM (− 1). Meropenem MICs for carbapenemase-harboring *P. aeruginosa* were > 8 μg/ mL.

All isolates were stored in skim milk (Becton Dickinson, Sparks, MD) at − 80 °C and subcultured to Trypticase soy agar with 5% sheep blood plates (Becton Dickinson, Sparks, MD) prior to incubation. Isolates were incubated, without selective disc pressure, at 37 °C for 18–20 h prior to second subculture before testing.

### Evaluation of mCIM/eCIM

The mCIM test was conducted as previously described for *P. aeruginosa* [5, 13, 14]. Briefly, 2 mL of trypticase soy broth were inoculated with a 10-μL loopful of *P. aeruginosa*, vortexed, and a 10 μg meropenem disk (Becton Dickinson, Sparks, MD, LOT: 9065664 and 9186033) was placed into the mixture. This mixture incubated for 4 h (±15 min). Following incubation, the meropenem disk was removed from the tube and placed on a Mueller-Hinton agar plate that was lawned with a 0.5 McFarland suspension of *Escherichia coli* ATCC 25922. Simultaneously, the eCIM test was performed with the standard 5 mM EDTA concentration, as well as doubling EDTA concentrations (i.e. 10 mM, 20 mM, and 40 mM). Isolates were run in duplicate at each concentration and zone diameters of inhibition were measured by two independent investigators. eCIM was interpreted as positive (detection of metallo-β-lactamase) if the zone of inhibition diameter increases by ≥5 mm compared with the isolate’s mCIM reading and was considered negative if the zone of inhibition diameter was ≤4 mm.

Data analysis was conducted in SPSS (IBM. Armonk, NY). Isolates’ phenotypic tests were compared to the genotypic standard. An isolate was defined as eCIM true positive if the phenotypic test matched an MBL-positive genotype or eCIM true negative if the phenotype matched a serine carbapenemase-positive genotype. eCIM false-positive results were defined as a phenotype indicating MBL production (eCIM positive) in isolates with negative genotypic findings and an eCIM false-negative result was defined as a negative phenotypic result (eCIM negative) despite presence of an MBL gene.

### Establishment of controls

Wild type *P. aeruginosa isolate* (ATCC 27853) served as a carbapenemase mCIM control. In order to establish appropriate and publically-accessible eCIM controls for use with *P. aeruginosa*, one KPC-harboring isolate (#0441) and one VIM-harboring *P.aeruginosa* isolate (#0444) from the CDC and FDA-AR isolate bank were utilized. Quality control testing was performed on each testing day.

## Data Availability

The datasets used and/or analysed during the current study are available from the corresponding author on reasonable request.

## Abbreviations

mCIM: Modified carbapenem inactivation method
eCIM: EDTA-modified carbapenem inactivation method
CLSI: Clinical and Laboratory Standards Institute
MBL: Metallo-β-lactamase
NDM: New Delhi metallo-β-lactamase
VIM: Verona integron-encoded metallo-β-lactamase
IMP: Imipenemase
KPC: *Klebsiella pneumoniae* carbapenemase
WT: Wild type
CDC: Centers for Disease Control
FDA-AR: Federal Drug and Administration Antibiotic Resistance Bank
